# Bacterial Extracellular Vesicles in Oncology: Molecular Mechanisms and Future Clinical Applications

**DOI:** 10.3390/cancers17111774

**Published:** 2025-05-26

**Authors:** Piyush Bhanu, Andrew K. Godwin, Shahid Umar, Diane E. Mahoney

**Affiliations:** 1 Bioengineering Program, School of Engineering, University of Kansas, Lawrence, KS 66045, USA; 2 Department of Pathology and Laboratory Medicine, University of Kansas Medical Center, Kansas City, KS 66160, USA; agodwin@kumc.edu; 3 The Kansas Institute for Precision Medicine, University of Kansas Medical Center, Kansas City, KS 66160, USA; 4 The University of Kansas Cancer Center, University of Kansas Medical Center, Kansas City, KS 66160, USA; 5 Department of Surgery, University of Kansas Medical Center, Kansas City, KS 66103, USA; sumar@kumc.edu; 6 School of Nursing, University of Kansas Medical Center, Kansas City, KS 66160, USA; dmahoney@kumc.edu

**Keywords:** bacterial extracellular vesicles, cancer progression, tumor microenvironment, immune modulation, angiogenesis, bacterial EV-based biomarkers, tumor management

## Abstract

Bacterial extracellular vesicles (BEVs) have emerged as pivotal mediators of host–microbe interactions, profoundly influencing cancer biology. These nanoscale vesicles, produced by both Gram-positive and Gram-negative bacteria, carry diverse biomolecular cargo such as proteins, lipids, nucleic acids, and metabolites. BEVs play dualistic roles in tumor promotion and suppression by modulating the tumor microenvironment, immune responses, and genetic regulation. This review synthesizes the current understanding of BEVs in various cancers, including gastrointestinal, ovarian, breast, lung, brain, and renal malignancies. BEVs are highlighted for their potential as diagnostic biomarkers, prognostic indicators, and therapeutic agents, including their applications in immunotherapy and advanced engineering for precision medicine. Challenges such as heterogeneity, standardization, and clinical scalability are critically analyzed, with case examples providing actionable insights. Future directions emphasize interdisciplinary collaborations, emerging technologies, and the integration of BEV-based tools into clinical workflows. This review underscores the transformative potential of BEVs in advancing cancer diagnostics and therapeutics, paving the way for innovations in precision oncology.

## Simple Summary

This review explores the emerging role of bacterial extracellular vesicles (BEVs) in cancer biology. These nanosized particles, released by both Gram-positive and negative bacteria, carry a range of biomolecules that can influence cancer progression by modulating the immune system, gene expression, and the tumor microenvironment. This paper examines how BEVs contribute to both tumor growth and suppression across several cancer types. It also highlights their potential as non-invasive tools for cancer diagnosis, prognosis, and therapy. Key challenges in standardization and clinical application are discussed, along with future directions for integrating BEV-based strategies into precision oncology.

## 1. Introduction

**Figure 1:**
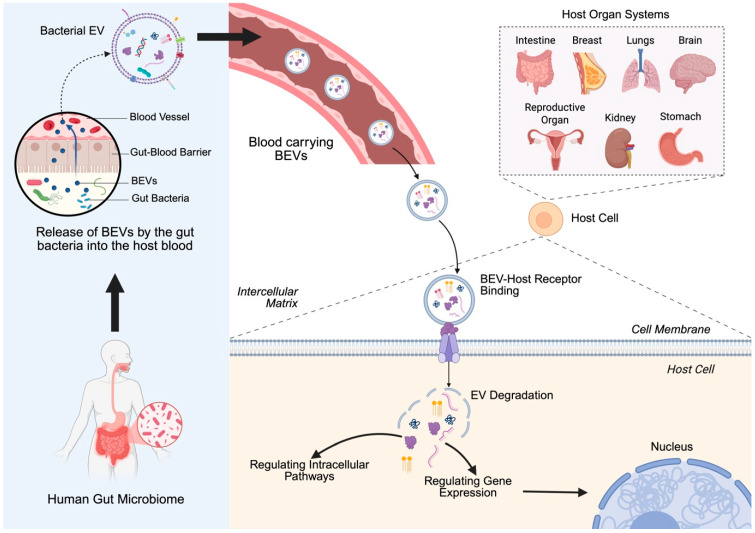
Bacterial extracellular vesicle (BEV) transport from the gut to host target cells: BEVs released by the gut microbiome cross the gut–blood barrier and enter systemic circulation. These vesicles can travel through the bloodstream and reach distant host organs. Upon arrival, BEVs bind to host cell receptors, facilitating their uptake and degradation, leading to intracellular signaling and gene regulation. Created in BioRender. https://BioRender.com/2bn4rhx (accessed on 4 April 2025) Pathak, H. [8].

Bacterial extracellular vesicles (BEVs) have emerged as key mediators in host–microbe interactions [1]. These nanoscale vesicles, produced by both Gram-positive and Gram-negative bacteria, carry a diverse cargo of biomolecules, including proteins, lipids, nucleic acids, and metabolites [2]. BEVs play pivotal roles in bacterial communication, biofilm formation, and modulation of host responses. Recent studies have unveiled their involvement in a variety of pathological conditions, including cancer [3]. By influencing processes such as immune modulation, genetic regulation, and chronic inflammation, BEVs have been implicated in both tumor promotion and suppression [4,5]. Thus, the exploration of BEVs has gained increasing attention in the fields of oncology. The potential of BEVs as biomarkers and therapeutic agents further underscores their relevance in cancer research [6]. Unlike mammalian-derived extracellular vesicles, BEVs have distinct lipid compositions, immunogenic profiles, and horizontal gene transfer capabilities, making them unique players in cancer pathogenesis [7]. Their ability to traverse biological barriers such as the gut and deliver functional cargo to host cells positions them as promising tools for early diagnosis, prognostic evaluation, and targeted therapy (Figure 1). However, understanding the precise mechanisms through which BEVs influence cancer progression remains a significant challenge, necessitating comprehensive and multidisciplinary investigations.

This review aims to provide a detailed examination of the role of BEVs in human cancers, with a focus on their biological properties, mechanisms of action, and applications in tumor management (Figure 1). By integrating insights from recent studies, we highlight the dualistic nature of BEVs in cancer biology and discuss their potential to transform cancer diagnostics and therapeutics. Through this synthesis, we hope to inspire further research into the untapped potential of BEVs that perpetuate innovative translational applications in clinical oncology.

## 2. Bacterial Extracellular Vesicles (BEVs)

### 2.1. Biology of Bacterial Extracellular Vesicles

**Figure 2:**
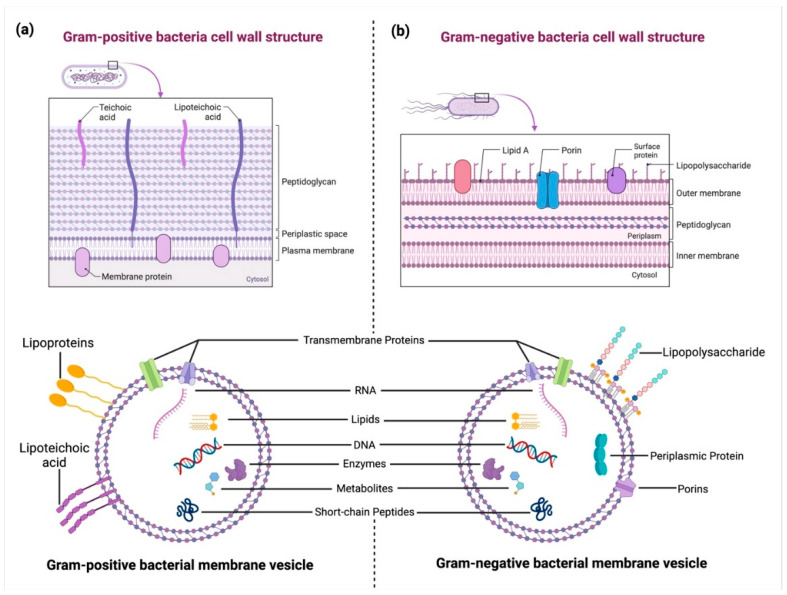
Structural differences between Gram-positive and Gram-negative bacterial cell walls and their extracellular vesicles (BEVs): (**a**) A Gram-positive bacterial cell wall consists of a thick peptidoglycan layer embedded with teichoic and lipoteichoic acids (LTA). BEVs from Gram-positive bacteria originate from the cytoplasmic membrane and carry biomolecules such as RNA, DNA, lipids, enzymes, metabolites, and proteins. (**b**) A Gram-negative bacterial cell wall features an outer membrane with lipopolysaccharides (LPS), porins, and surface proteins, enclosing a periplasmic space. Gram-negative bacteria release outer membrane vesicles (OMVs) that contain periplasmic proteins, nucleic acids, lipids, and enzymes, playing key roles in host–pathogen interactions. Created in BioRender. https://BioRender.com/2bn4rhx (accessed on 4 April 2025) Pathak, H. [8].

BEVs are nanosized, membrane-enclosed structures released by both Gram-positive and Gram-negative bacteria (Figure 2) [2]. These vesicles range in size from 20 to 400 nanometers and are formed through the outward budding of the bacterial membrane. While Gram-negative bacteria release outer membrane vesicles (OMVs) originating from the outer membrane, Gram-positive bacteria, despite lacking an outer membrane, also secrete vesicles through mechanisms that are not yet fully understood [9]. However, Gram-positive BEVs are proposed to bud directly from the cytoplasmic membrane (Figure 2a) [10].

### 2.2. Molecular Composition of BEVs

BEVs carry a complex cargo that mirrors the composition of their parent bacteria [11]. This includes proteins, lipids, polysaccharides, nucleic acids (DNA and RNA), and metabolites. Proteins within BEVs can serve as virulence factors, enzymes, or signaling molecules, while lipids contribute to their structural integrity and interaction with host membranes. Additionally, nucleic acids in BEVs have been implicated in horizontal gene transfer and modulation of host gene expression [9]. Table 1 provides a brief description of the known function of BEV molecular cargo specifically in cancer.

### 2.3. Mechanisms of BEV Formation

The biogenesis of BEVs involves multiple processes, including membrane remodeling and turgor pressure-driven extrusion (Figure 2). In Gram-negative bacteria, OMVs form through the decoupling of the outer membrane from the peptidoglycan layer (Figure 2b) [29]. In Gram-positive bacteria, vesicle formation may be facilitated by localized disruptions in the cell wall or by enzymatic activity [30]. Environmental factors such as stress, nutrient availability, and antibiotic exposure can influence the rate and composition of BEV production [31].

### 2.4. Isolation and Characterization of BEVs

The study of BEVs requires robust methods for their isolation and analysis. Ultracentrifugation, density gradient centrifugation, and size-exclusion chromatography are commonly employed techniques for isolating BEVs [32]. Characterization typically involves electron microscopy, nanoparticle tracking analysis (NTA), and proteomic or genomic analyses to elucidate their size, morphology, and molecular composition [33]. Standardization of these methods remains a challenge, particularly in the context of clinical applications.

## 3. Role of BEVs in Cancer

**Figure 3:**
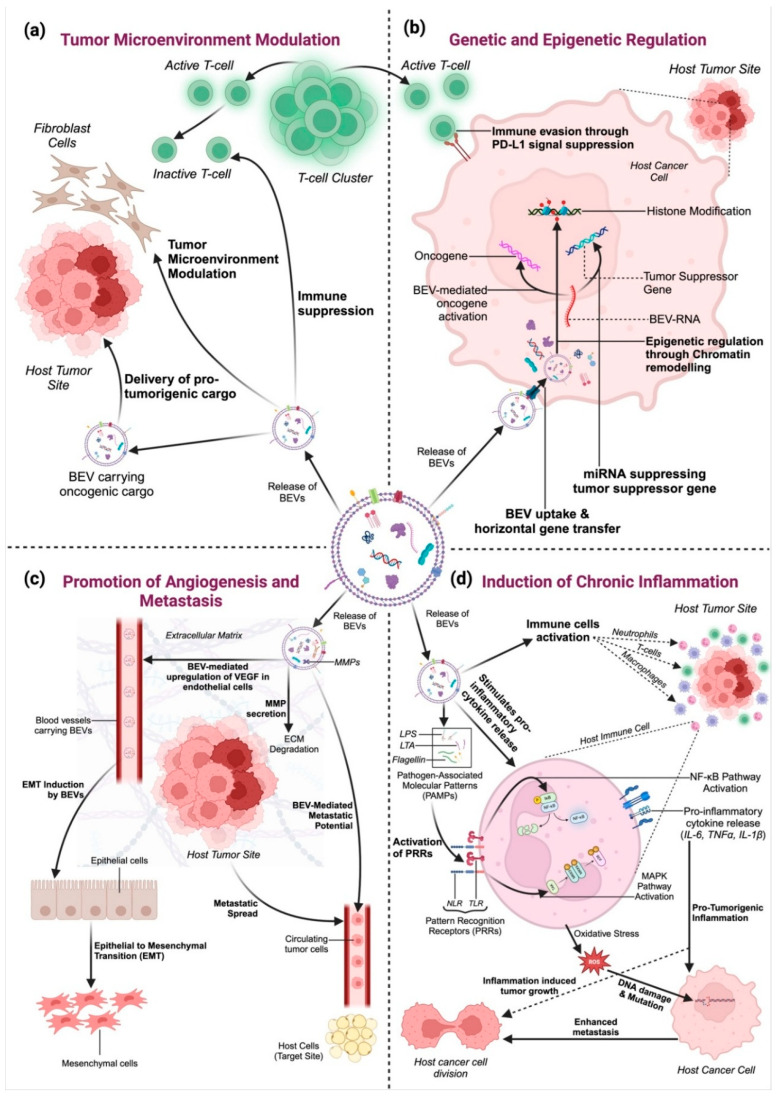
The multifaceted role of bacterial extracellular vesicles (BEVs) in tumor progression: (**a**) Tumor Microenvironment Modulation: BEVs deliver oncogenic cargo to immune cells and fibroblasts, suppressing immune responses and supporting tumor growth. (**b**) Genetic and Epigenetic Regulation: BEVs transfer RNA, DNA, and regulatory proteins to tumor cells, promoting oncogene activation and suppressing tumor suppressors through genetic and epigenetic modifications. (**c**) Angiogenesis and Metastasis Promotion: BEVs induce VEGF expression, enhancing blood vessel formation, and facilitate metastasis by delivering matrix metalloproteinases (MMPs) for ECM degradation and promoting epithelial-to-mesenchymal transition (EMT). (**d**) Induction of Chronic Inflammation: BEVs carry pathogen-associated molecular patterns (PAMPs), such as LPS and peptidoglycan, which activate host immune pathways (e.g., NF-κB and MAPK), leading to chronic inflammation, oxidative stress, and DNA damage. Created in BioRender. https://BioRender.com/2bn4rhx (accessed on 4 April 2025) Pathak, H. [8].

Understanding the biology of BEVs is fundamental to exploring their roles in human health and disease. Their diverse cargo and ability to interact with host cells make them potent mediators of bacterial influence, with significant implications for cancer biology and beyond. Cancer remains one of the leading causes of mortality worldwide, with an estimated 10 million deaths attributed to the disease in 2022 alone [34]. Despite significant advancements in early detection and treatment strategies, the complexity of cancer biology continues to pose challenges for effective management. A critical aspect of cancer progression is the interaction between tumor cells and their microenvironment, a dynamic interplay that involves the host immune system, stromal components, and microbial communities (Figure 3) [35,36]. BEVs play a multifaceted role in cancer progression by modulating key biological processes that influence tumor development and metastasis (Figure 3) [22]. Their ability to interact with host cells and the tumor microenvironment has positioned BEVs as both facilitators and regulators of cancer progression [7]. This section delves into the mechanisms through which BEVs contribute to cancer biology.

### 3.1. Tumor Microenvironment Modulation

BEVs significantly influence the tumor microenvironment by interacting with cancer cells, immune cells, and stromal components (Figure 3a) [23,27]. They can promote tumor growth by delivering pro-tumorigenic molecules such as oncogenic proteins, RNA, and lipids [2]. BEVs also modulate immune responses, often creating an immunosuppressive environment that allows tumors to evade immune surveillance (Figure 3a).

For instance, BEVs from *Fusobacterium nucleatum* have been shown to promote an immunosuppressive environment in colorectal cancer by polarizing macrophages to an M2 phenotype, which supports tumor growth and immune escape (Figure 3a) [37]. Additionally, BEVs can inhibit dendritic cell maturation, reduce antigen presentation, and limit anti-tumor immunity (Figure 3a) [38]. In breast cancer, BEVs can also enhance fibroblast activation, transforming them into cancer-associated fibroblasts (CAFs) that secrete growth factors supporting tumor proliferation (Figure 3a) [39]. Furthermore, BEVs can deliver immune checkpoint molecules such as PD-L1, which directly suppress T-cell activation and promote immune evasion (Figure 3a) [40,41].

### 3.2. Genetic and Epigenetic Regulation

BEVs facilitate the horizontal transfer of genetic material, including DNA and RNA, to host cells, thereby influencing gene expression and cellular behavior (Figure 3b) [40]. MicroRNAs (miRNAs) carried by BEVs have been shown to regulate key oncogenic pathways, contributing to cancer cell proliferation, migration, and survival [42,43].

For instance, BEVs from *Helicobacter pylori* have been shown to deliver the virulence factor CagA, which directly enters host gastric epithelial cells and modulates host signaling pathways, promoting oncogenesis (Figure 3b) [44]. Similarly, BEVs from *Fusobacterium nucleatum* in colorectal cancer have been reported to carry miR-21, an oncogenic miRNA that suppresses tumor suppressor pathways and enhances cell proliferation (Figure 3b) [37]. Additionally, BEVs can modulate host cell epigenetics by delivering enzymes and other molecules that alter DNA methylation and histone modification patterns (Figure 3b) [45].

Beyond direct gene regulation, BEVs also transport other regulatory RNAs, such as small RNAs (sRNAs) and long non-coding RNAs (lncRNAs), which can further modulate host cell transcriptomes (Figure 3b). These molecules may interfere with host RNA processing, leading to the suppression of tumor suppressors or the activation of oncogenes [46]. Moreover, BEVs have been reported to deliver bacterial DNA methyltransferases (DNMTs) and histone-modifying enzymes that influence chromatin remodeling, potentially altering gene accessibility and transcriptional activity in recipient cells (Figure 3b) [47]. For example, BEVs from *Pseudomonas aeruginosa* have been shown to induce global histone deacetylation in lung epithelial cells, creating a permissive environment for tumor development (Figure 3b) [48]. Such epigenetic modifications can promote immune evasion, sustain chronic inflammation, and contribute to therapy resistance in cancer [49]. The ability of BEVs to regulate both genetic and epigenetic landscapes highlights their role as crucial mediators in tumor progression. Therefore, investigating their cargo and mechanisms of action may open new avenues for biomarker discovery and targeted therapeutic interventions in cancer.

### 3.3. Promotion of Angiogenesis and Metastasis

The ability of tumors to grow and metastasize depends on angiogenesis, the formation of new blood vessels. Emerging studies suggest that BEVs may influence angiogenic pathways, potentially via modulation of VEGF expression, thereby affecting endothelial cell behavior, although direct mechanistic evidence remains limited (Figure 3c) [29,50].

For instance, BEVs from *Pseudomonas aeruginosa* have been shown to directly induce VEGF expression in endothelial cells, promoting angiogenesis and enhancing blood vessel formation (Figure 3c) [48]. Similarly, BEVs derived from *Fusobacterium nucleatum* in colorectal cancer can carry pro-angiogenic factors, further promoting vascularization (Figure 3c) [37]. Additionally, as shown in Figure 3c, BEVs can enhance cancer cell invasiveness and metastatic potential by modulating the extracellular matrix and facilitating epithelial-to-mesenchymal transition (EMT) [47]. BEVs also enhance metastatic potential by carrying MMPs that degrade the extracellular matrix (ECM), creating pathways for cancer cell invasion (Figure 3c) [51]. For example, BEVs from *Helicobacter pylori* have been shown to induce EMT in gastric cancer cells, facilitating metastatic spread (Figure 3c) [44].

### 3.4. Induction of Chronic Inflammation

Chronic inflammation is a well-established hallmark of cancer, and BEVs are potent mediators of inflammatory responses (Figure 3d). BEVs can stimulate the release of pro-inflammatory cytokines and chemokines, fostering an inflammatory environment that supports tumor initiation and progression. Furthermore, as shown in Figure 3d, BEVs from pathogenic bacteria have been linked to the activation of pathways such as NF-κB and MAPK, which are critical in inflammation-induced carcinogenesis [52].

For instance, BEVs from *Escherichia coli* have been shown to carry lipopolysaccharides (LPS), potent pathogen-associated molecular patterns (PAMPs) that activate Toll-like receptors (TLRs) on macrophages, leading to NF-κB activation and pro-inflammatory cytokine release (Figure 3d) [53]. Similarly, BEVs from *Helicobacter pylori* can deliver peptidoglycan, which activates Nod-like receptors (NLRs) in host cells, further enhancing pro-inflammatory signaling (Figure 3d) [44].

Beyond cytokine induction, BEVs can promote persistent immune activation by carrying PAMPs, such as LPS, peptidoglycans, and flagellin, which interact with host pattern recognition receptors (PRRs) like TLRs and Nod-like receptors (NLRs) (Figure 3d) [12,53,54]. This interaction triggers downstream signaling cascades that sustain chronic inflammation, leading to the recruitment and activation of immune cells, including macrophages, neutrophils, and dendritic cells. The resulting inflammatory microenvironment promotes oxidative stress and DNA damage, both of which contribute to tumorigenesis [55]. By orchestrating these processes, BEVs function as key modulators of cancer progression, highlighting their dualistic nature as both contributors to tumor development and potential targets for therapeutic intervention (Figure 3).

## 4. BEVs Across Human Cancer Types

BEVs exhibit diverse roles in various human cancers, influencing tumor initiation, progression, and metastasis in a cancer-specific manner. This section explores their involvement in different types of cancers and highlights their unique contributions to each. Table 2 provides a summary of the role of BEVs in cancer pathogenesis by type. However, the strength of evidence varies across cancer types, with some roles supported by limited or preliminary studies.

### 4.1. Colorectal Cancer (CRC)

Colorectal cancer (CRC) is a leading cause of cancer-related deaths globally, driven by genetic, epigenetic, and microbial influences [65]. Recent studies highlight the role of BEVs in CRC development, particularly in tumor initiation, metastasis, and therapy resistance. BEVs from pathogenic bacteria contribute to CRC progression by inducing DNA damage, modulating immune responses, and promoting a pro-tumorigenic microenvironment [56]. Their potential as diagnostic biomarkers further underscores their significance in CRC research.

Tumor Initiation and Progression: BEVs from pathogenic bacteria such as *Escherichia coli* and *Fusobacterium nucleatum* have been implicated in CRC development. *E. coli* strains producing colibactin can induce DNA double-strand breaks in colonic epithelial cells, leading to genomic instability and tumorigenesis. *F. nucleatum* BEVs facilitate immune evasion by modulating T-cell responses, creating an inflammatory microenvironment conducive to cancer progression [56].

Additionally, BEVs from *F. nucleatum* carry miR-21, which directly suppresses tumor suppressor genes like PTEN, promoting cancer cell proliferation [66]. BEVs also enhance immune evasion by polarizing macrophages toward an M2 phenotype, creating an immunosuppressive environment [48].

Metastasis: BEVs contribute to CRC metastasis by promoting EMT, enhancing cell motility, and preparing pre-metastatic niches in distant organs. They transfer oncogenic proteins and RNAs that modulate signaling pathways involved in cell adhesion and migration [57]. BEVs from *F. nucleatum* have been shown to promote EMT through activation of Wnt/β-catenin signaling, enhancing cell migration. BEVs can also deliver MMPs, degrading the extracellular matrix (ECM) and creating a pro-metastatic environment [57].

Therapy Resistance: BEVs can mediate resistance to chemotherapy and targeted therapies in CRC. They transport drug efflux pumps, anti-apoptotic proteins, and regulatory RNAs that diminish drug efficacy, posing challenges to effective treatment [57,67]. For example, BEVs from *E. coli* have been shown to carry ABC transporters, reducing drug accumulation within tumor cells and diminishing chemotherapy efficacy. Additionally, BEVs can deliver anti-apoptotic proteins such as Bcl-2, which protect tumor cells from drug-induced apoptosis [67].

Diagnostic and Prognostic Potential: The molecular cargo of BEVs reflects the physiological state of their cells of origin, making them valuable as non-invasive biomarkers for CRC detection and monitoring. Circulating BEVs in patient blood samples have been studied for their potential to provide insights into tumor dynamics and treatment responses [68,69]. Specifically, BEVs carrying miRNAs such as miR-92a and miR-21 have been identified in CRC patient blood samples, offering a non-invasive approach for early detection and monitoring [69]. Elevated levels of BEV-associated PD-L1 have also been linked to poor patient outcomes in CRC [68].

### 4.2. Gastric Cancer (GC)

Gastric cancer (GC) remains one of the leading causes of cancer-related mortality worldwide, often associated with *Helicobacter pylori* infection [70]. BEVs from *H. pylori* and other gastric microbiota play a crucial role in GC pathogenesis by delivering virulence factors, modulating immune responses, and altering host cellular pathways. These vesicles contribute to tumor progression, metastasis, and therapy resistance, highlighting their significance in GC development [51]. Additionally, BEVs are being investigated as potential biomarkers for early detection and as innovative therapeutic delivery systems, offering promising avenues for GC diagnosis and treatment [71].

Tumor Initiation and Progression: *Helicobacter pylori* BEVs are central to GC pathogenesis. They deliver virulence factors such as CagA and VacA into gastric epithelial cells, disrupting cellular functions and activating oncogenic pathways like ERK and PI3K/AKT, leading to malignant transformation [58]. BEVs from *H. pylori* can also deliver peptidoglycan, which activates Nod-like receptors (NLRs) in host cells, promoting pro-inflammatory signaling and supporting tumor progression [71]. Additionally, BEVs have been shown to carry outer membrane vesicle proteins (OMPs) that directly interact with epithelial cells, enhancing cell proliferation and survival [58].

Metastasis: BEVs facilitate GC metastasis by remodeling the tumor microenvironment, promoting angiogenesis, and inducing EMT. They carry matrix metalloproteinases (MMPs) and pro-angiogenic factors that degrade the extracellular matrix and support new blood vessel formation, aiding in tumor spread [51,67]. BEVs from *H. pylori* can promote EMT by delivering miR-135b, which suppresses E-cadherin expression, enhancing cell migration and invasion [51]. Furthermore, BEVs also enhance angiogenesis by inducing VEGF expression in endothelial cells, promoting new blood vessel formation and facilitating metastatic spread [68].

Drug Resistance: In GC, BEVs contribute to chemoresistance by transferring molecules that inhibit apoptosis and enhance drug efflux. This includes the delivery of specific RNAs and proteins that alter cellular responses to chemotherapy, complicating treatment strategies [24]. BEVs from *H. pylori* can deliver miR-155, which downregulates pro-apoptotic proteins such as Bax, promoting cell survival even under chemotherapeutic stress. Additionally, they can carry efflux pumps that reduce intracellular drug concentrations, further diminishing chemotherapy efficacy [24].

Diagnostic and Therapeutic Applications: BEVs in GC patients’ bodily fluids are being explored as biomarkers for early detection and as vehicles for targeted therapy. Their unique molecular signatures offer opportunities for developing non-invasive diagnostic tools and novel therapeutic approaches [14,68]. Circulating BEVs containing *H. pylori*-specific miRNAs (e.g., miR-223 and miR-155) have been identified in GC patients and can serve as potential non-invasive biomarkers for early detection [70]. Moreover, BEVs are being engineered as therapeutic carriers to deliver anti-tumor drugs directly to cancer cells, enhancing targeted treatment with reduced side effects [14].

Understanding the multifaceted roles of BEVs in gastrointestinal cancers is crucial for developing innovative diagnostic and therapeutic strategies. Ongoing research into the molecular mechanisms of BEV interactions within the tumor microenvironment holds promise for advancing precision oncology in CRC and GC.

### 4.3. Breast Cancer

Breast cancer is the most common malignancy in women globally, with a complex etiology influenced by genetic, hormonal, and environmental factors [72]. Recent studies suggest that BEVs contribute to breast cancer progression by modulating gene expression, immune evasion, and therapy resistance. BEVs derived from the microbiota of the urine samples of women with and without breast cancer carry oncogenic miRNAs, enzymes, and signaling molecules that enhance tumor growth, facilitate metastasis, and alter immune responses [37]. Additionally, their role in therapy resistance highlights their potential as targets for improving treatment outcomes. Beyond pathogenesis, BEVs are being explored as non-invasive biomarkers for early detection and as innovative therapeutic carriers, offering promising applications in breast cancer management.

Tumor Progression and Immune Modulation: BEVs derived from bacteria in the breast tissue microenvironment carry oncogenic miRNAs such as miR-21 and miR-155, which target tumor suppressor genes, enabling unchecked cell proliferation [44]. Additionally, BEVs interact with immune cells to suppress anti-tumor responses. For example, they can induce the polarization of macrophages toward a pro-tumorigenic M2 phenotype, which fosters an immune-suppressive microenvironment [73]. BEVs have also been shown to inhibit dendritic cell maturation, reducing antigen presentation and weakening anti-tumor immunity [44]. In addition, BEVs can carry PD-L1, which directly suppresses T-cell activation, promoting immune evasion [74].

Metastasis Facilitation: BEVs play a critical role in the metastatic cascade of breast cancer by interacting with the extracellular matrix (ECM). They carry enzymes like MMPs that degrade ECM components, facilitating cancer cell invasion and migration [39]. Furthermore, BEVs promote EMT, a crucial step in metastasis, by delivering signaling molecules such as transforming growth factor-beta (TGF-β) [28,74]. BEVs from the breast tissue microbiota can also deliver miR-10b, a well-known metastatic miRNA, which enhances cell migration and invasion by targeting E-cadherin and promoting EMT [75].

Therapy Resistance: Resistance to chemotherapeutic agents such as tamoxifen has been linked to BEV-mediated signaling pathways. BEVs transport molecules that activate the PI3K/AKT and ERK pathways, reducing the efficacy of therapies targeting these mechanisms. Moreover, BEVs influence the redox balance within breast cancer cells by modulating reactive oxygen species (ROS) levels, which can contribute to apoptosis resistance [41]. Specifically, BEVs can deliver anti-apoptotic proteins such as Bcl-2, which inhibits cell death, and miR-221, which downregulates pro-apoptotic factors like Bax, enhancing cell survival [41].

Clinical Implications: BEVs in breast cancer patients’ blood and tissue samples have shown potential as biomarkers for early detection and as indicators of metastatic potential. Studies have also explored engineered BEVs as therapeutic delivery vehicles, particularly for RNA-based interventions targeting oncogenic pathways [75,76].

### 4.4. Lung Cancer

Lung cancer is the leading cause of cancer-related deaths worldwide, with environmental, genetic, and microbial factors contributing to its pathogenesis [20]. Recent evidence suggests that BEVs play a role in lung cancer progression by modulating chronic inflammation, facilitating metastasis, and promoting therapy resistance [38]. BEVs from lung-resident and pathogenic bacteria influence the tumor microenvironment through immune evasion, oxidative stress, and extracellular matrix remodeling [27]. Additionally, their potential as biomarkers for early detection and as carriers for targeted therapies highlights their relevance in lung cancer diagnosis and treatment strategies.

Tumor Progression and Chronic Inflammation: BEVs from lung-resident or pathogenic bacteria modulate chronic inflammation, a critical driver of tumorigenesis in lung cancer. For instance, BEVs act as pro-inflammatory mediators where BEVs enriched with interleukins (e.g., IL-6) and tumor necrosis factor-alpha (TNF-α) activate nuclear factor kappa B (NF-κB) pathways in epithelial cells, fostering an inflammatory microenvironment conducive to carcinogenesis [38,77]. BEVs also deliver ROS or modulate host ROS production, leading to DNA damage and mutations that initiate tumorigenesis [48,78]. Additionally, BEVs from *Pseudomonas aeruginosa* carry lipopolysaccharides (LPS), which activate Toll-like receptors (TLRs) on lung epithelial cells, triggering NF-κB signaling and promoting sustained inflammation [38]. BEVs can also enhance oxidative stress by downregulating antioxidant enzymes such as superoxide dismutase (SOD), increasing ROS levels, and promoting DNA damage [79].

Metastasis Facilitation: BEVs contribute to lung cancer metastasis through pre-metastatic niche formation where BEVs from pathogens such as *Pseudomonas aeruginosa* carry lipopolysaccharides (LPS) and other factors that prime distant tissues, preparing them for metastatic colonization [7]. BEVs also trigger extracellular matrix remodeling through enzymes like MMPs transported by BEVs that degrade the extracellular matrix, facilitating cancer cell invasion and migration [59].

Therapy Resistance: Resistance to therapies, including chemotherapy and targeted treatments, is mediated by BEVs via oncogenic cargo delivery [27]. BEVs transfer miRNAs and proteins that activate survival pathways such as PI3K/AKT and MEK/ERK, conferring resistance to apoptosis [79,80]. BEVs might also lead to drug efflux enhancement, as they carry efflux pump proteins that decrease intracellular drug concentrations, rendering chemotherapy less effective [8]. BEVs from *Pseudomonas aeruginosa* can carry miR-21, which inhibits pro-apoptotic proteins such as Bax, promoting cell survival even under chemotherapeutic stress [79]. Additionally, BEVs can deliver miR-155, which activates the PI3K/AKT pathway, enhancing resistance to apoptosis [81].

Immune Modulation: BEVs manipulate immune responses to benefit tumor survival through T-cell suppression as BEVs deliver immune checkpoint ligands like PD-L1, reducing T-cell activity and enabling immune evasion. By inducing macrophage polarization to the M2 phenotype, BEVs create an immunosuppressive microenvironment that supports tumor growth [27,81]. BEVs can also suppress natural killer (NK) cell activity by delivering transforming growth factor-beta (TGF-β), reducing their cytotoxicity against tumor cells [82]. Additionally, BEVs carrying miR-23a have been shown to suppress T-cell activation by targeting CD8+ T-cell signaling pathways [27].

Diagnostic and Therapeutic Applications: BEVs hold promise as tools for non-invasive diagnostics and targeted therapies. BEVs isolated from bronchoalveolar lavage fluid or blood samples contain lung cancer-specific proteins and miRNAs, making them valuable biomarkers for early detection and disease monitoring [82]. Engineered BEVs are being developed as therapeutic delivery vehicles to deliver anti-cancer agents, including small interfering RNAs (siRNAs) targeting oncogenes such as KRAS, directly to tumor sites [8,83].

### 4.5. Brain Cancer

Brain cancer, particularly glioblastoma, is one of the most aggressive malignancies, characterized by rapid progression and poor prognosis [84]. Emerging evidence suggests that BEVs contribute to brain tumor development by modulating the tumor microenvironment, promoting immune evasion, and enhancing therapy resistance [60]. BEVs can cross the blood–brain barrier, carrying oncogenic factors and pro-inflammatory molecules that support tumor growth and survival [85]. Additionally, their potential as non-invasive biomarkers and therapeutic carriers opens new avenues for early detection and targeted treatment strategies in brain cancer.

Tumor Microenvironment Modulation: BEVs can cross the blood–brain barrier, delivering oncogenic factors such as epidermal growth factor receptor variant III (EGFRvIII) to glioblastoma cells. This contributes to enhanced cell proliferation, invasion, and resistance to apoptosis [86]. Additionally, BEVs carry pro-inflammatory molecules like LPS and cytokines, which activate microglial cells and create a tumor-supportive inflammatory environment [87]. BEVs from *Escherichia coli* have also been shown to deliver outer membrane vesicle proteins that trigger microglial activation, thereby promoting neuroinflammation [87]. Additionally, BEVs can deliver oncogenic miRNAs (e.g., miR-21 and miR-155) that directly enhance glioblastoma cell proliferation and invasion [86].

Immune Evasion: BEVs play a role in dampening the anti-tumor immune response in the brain. They carry immune checkpoint molecules such as programmed death-ligand 1 (PD-L1), which interact with T-cells and reduce their activity, allowing glioblastoma cells to evade immune surveillance [88]. BEVs also promote the recruitment of regulatory T-cells (Tregs) to the tumor site, further suppressing immune responses [89]. In addition, BEVs can deliver transforming growth factor-beta (TGF-β), which suppresses cytotoxic T-cell function and promotes the expansion of regulatory T-cells (Tregs) in the tumor microenvironment [89].

Therapy Resistance: Resistance to standard therapies, including radiation and chemotherapy, is a hallmark of glioblastoma. BEVs contribute to this by transferring molecules such as anti-apoptotic proteins, drug efflux pumps, and regulatory RNAs to tumor cells, enhancing their survival under therapeutic stress. Moreover, BEVs can activate pathways like PI3K/AKT and MAPK, which are associated with therapy resistance [80]. Specifically, BEVs carrying miR-21 can downregulate PTEN, a tumor suppressor, enhancing PI3K/AKT pathway activation and promoting therapy resistance. BEVs can also deliver miR-221, which inhibits pro-apoptotic factors, further supporting cell survival under therapeutic stress [80].

Diagnostic and Therapeutic Implications: BEVs in cerebrospinal fluid (CSF) and blood samples of glioblastoma patients contain specific biomarkers, such as EGFRvIII and pro-inflammatory cytokines, which could aid in early diagnosis and monitoring. Engineered BEVs are also being explored as delivery vehicles for targeted therapies, including RNA-based treatments and immune checkpoint inhibitors [60,86].

### 4.6. Renal and Bladder Cancers

Renal and bladder cancers are among the most common malignancies of the urinary tract, often influenced by chronic inflammation and microbial interactions [90]. Recent studies suggest that BEVs, particularly those from uropathogenic *Escherichia coli* (UPEC) and other urinary tract pathogens, contribute to tumor progression by inducing inflammatory responses, modulating immune activity, and promoting therapy resistance [91]. BEVs facilitate epithelial-to-mesenchymal transition (EMT), metastasis, and drug resistance mechanisms, highlighting their significance in disease progression [92]. Additionally, their presence in urine as potential biomarkers makes them promising tools for early cancer detection and targeted therapeutic applications.

Tumor Progression and Immune Modulation: BEVs derived from uropathogenic *Escherichia coli* (UPEC) and other urinary tract pathogens have been shown to induce chronic inflammation, a key driver of tumor initiation in renal and bladder tissues [91]. BEVs carry inflammatory mediators such as LPS, cytokines, and bacterial DNA/RNA, which activate Toll-like receptor (TLR) signaling pathways in epithelial and immune cells. This creates a pro-tumorigenic microenvironment characterized by increased cell proliferation and angiogenesis [93]. Additionally, BEVs from UPEC can deliver peptidoglycan, which activates Nod-like receptors (NLRs) in epithelial cells, further amplifying pro-inflammatory signaling [91]. BEVs carrying miR-155 have also been shown to downregulate immune checkpoints, promoting immune evasion in bladder cancer [93].

Metastasis Facilitation: BEVs play a critical role in the metastatic cascade by facilitating EMT, a process essential for cancer cell invasion and migration. BEVs deliver molecules such as transforming growth factor-beta (TGF-β) and MMPs, which degrade extracellular matrix components and promote cell motility [61]. Additionally, BEVs contribute to the formation of pre-metastatic niches by recruiting bone marrow-derived cells to distant tissues [94]. In addition, BEVs from UPEC can deliver miR-21, which promotes EMT by suppressing E-cadherin expression, enhancing cancer cell migration, and invasion [61]. BEVs can also carry integrins (e.g., ITGαV), which facilitate adhesion to distant metastatic sites [94].

Therapy Resistance: Resistance to standard therapies, including immunotherapy and targeted treatments, is a growing concern in renal and bladder cancers. BEVs transport molecules that upregulate survival pathways, inhibit apoptosis, and modulate drug efflux mechanisms, reducing the efficacy of therapeutic agents [93]. For example, BEVs carrying miRNAs such as miR-21 have been linked to enhanced resistance to cisplatin-based chemotherapy in bladder cancer [95].

Diagnostic and Therapeutic Implications: BEVs in urine samples from patients with renal and bladder cancers hold promise as non-invasive biomarkers for early detection and monitoring. Their molecular cargo, reflecting the tumor’s genetic and proteomic landscape, offers insights into disease progression and therapeutic response [93]. Additionally, engineered BEVs are being investigated as vehicles for delivering anti-cancer agents directly to tumor sites, minimizing systemic toxicity and enhancing treatment efficacy [96].

### 4.7. Ovarian Cancer

Ovarian cancer is a highly aggressive gynecological malignancy characterized by late-stage diagnosis and frequent recurrence [97]. Emerging evidence suggests that BEVs play a pivotal role in ovarian cancer progression by modulating immune responses, promoting angiogenesis, and facilitating metastasis. BEVs contribute to tumor immune evasion, chemoresistance, and inflammation-driven carcinogenesis, making them key players in the tumor microenvironment [62]. Additionally, their presence in bodily fluids highlights their potential as diagnostic biomarkers and therapeutic targets, offering promising avenues for precision medicine approaches in ovarian cancer management.

Immune Evasion and Suppression: BEVs can modulate immune responses in ovarian cancer, facilitating immune evasion and tumor growth. BEVs have been observed to carry immunomodulatory molecules that polarize macrophages towards an M2-like phenotype, known for its pro-tumorigenic functions [98]. This polarization suppresses the cytotoxic activity of T-cells and natural killer (NK) cells, creating an immunosuppressive microenvironment that favors tumor survival and progression [63].

Angiogenesis Promotion: Ovarian tumors rely heavily on angiogenesis to sustain their rapid growth and metastatic potential [99]. BEVs have been found to deliver pro-angiogenic factors such as VEGF and MMPs to endothelial cells, promoting the formation of new blood vessels. This neovascularization supports the growth of ovarian tumors and facilitates their dissemination to distant organs [62].

Chemoresistance Mechanisms: Chemoresistance remains a significant hurdle in the treatment of ovarian cancer. BEVs play a critical role in mediating resistance by transferring chemoresistance-associated molecules, such as drug efflux pumps and anti-apoptotic proteins, to cancer cells. Furthermore, BEVs can alter the expression of genes associated with drug metabolism and apoptosis, enabling cancer cells to withstand the cytotoxic effects of chemotherapy [100].

Metastasis Facilitation: The peritoneal dissemination of ovarian cancer cells is a hallmark of advanced-stage disease. BEVs enhance this metastatic spread by promoting EMT, a process critical for cancer cell invasion and migration. By delivering signaling molecules and miRNAs that modulate EMT-related pathways, BEVs facilitate the detachment of cancer cells from the primary tumor and their invasion into the peritoneal cavity [101].

Microbiome-Derived BEVs and Inflammation: The role of the microbiome in ovarian cancer is gaining attention, with BEVs emerging as critical mediators of microbiome–host interactions. BEVs from pathogenic bacteria in the genital or gastrointestinal tract can induce chronic inflammation, a known driver of carcinogenesis. These vesicles activate inflammatory pathways such as NF-κB and STAT3 in ovarian epithelial cells, promoting a pro-tumorigenic inflammatory environment [22].

Potential for Therapeutic Targeting: Given their multifaceted roles in ovarian cancer progression, BEVs present a promising target for therapeutic intervention. Strategies to inhibit BEV production, block their uptake by host cells, or neutralize their cargo are being explored [102]. Additionally, BEVs themselves could be harnessed as delivery vehicles for anti-cancer agents, leveraging their natural ability to target specific cells and tissues [103]. The involvement of BEVs in ovarian cancer highlights their importance as both contributors to disease progression and potential targets for innovative therapies. Ongoing research is needed to fully elucidate their mechanisms of action and to translate these findings into clinical applications.

## 5. Role of BEVs in Tumor Management

The translational potential of BEVs in tumor management is gaining significant attention. These vesicles offer unique advantages as diagnostic, prognostic, and therapeutic tools in oncology, owing to their ability to encapsulate and deliver biologically active molecules, interact with host cells, and traverse biological barriers [7]. This section discusses the applications of BEVs in tumor management, highlighting recent advancements and future prospects.

### 5.1. BEVs as Diagnostic Biomarkers

BEVs hold promise as non-invasive biomarkers for cancer diagnosis and monitoring. Their molecular cargo, which reflects the physiological state of their parent bacteria, can provide valuable insights into the tumor microenvironment. Advances in proteomics, transcriptomics, and metabolomics have enabled the identification of specific BEV-associated signatures linked to various cancers. For example, in colorectal cancer, BEV cargo profiling has revealed DNA adducts and RNA species linked to colibactin-producing *E. coli* strains [104]. Studies have demonstrated that serum BEVs carrying colibactin metabolites correlate strongly with tumor initiation in CRC patients. Advanced biosensor technologies, such as CRISPR-based diagnostics, are now employed to detect CRC-specific BEV markers in stool samples, further improving non-invasive diagnostic capabilities [105]. In lung cancer, beyond miR-21 and miR-210, BEVs have been found to carry specific lipids and proteins associated with oxidative stress and hypoxia in lung tumors [21]. These signatures are being validated as part of multiplex assays for early detection. Liquid biopsy platforms incorporating BEV analysis with bronchoalveolar lavage fluid have achieved sensitivities above 90%, enabling pre-symptomatic detection of lung malignancies [38]. In ovarian cancer, small EVs derived from serum samples carrying markers such as CA125, HE4, and tumor-associated miRNAs are being integrated into predictive models for ovarian cancer screening [106]. These models outperform traditional diagnostic methods in sensitivity and specificity. Microfluidic devices capable of isolating BEVs directly from ascitic fluid are in development, providing real-time insights into tumor heterogeneity and progression [32].

Recent innovations in microfluidics and biosensors have further enhanced the sensitivity and specificity of BEV-based diagnostics. For instance, integrated nano-sensor platforms can isolate and analyze BEVs from minute volumes of blood, enabling real-time monitoring of tumor dynamics [107]. Additionally, machine learning algorithms are being trained to identify cancer-specific BEV signatures, paving the way for automated and scalable diagnostic workflows [108]. Moreover, BEV diagnostics are being explored in combination with other liquid biopsy components, such as circulating tumor DNA (ctDNA) and exosomes, to provide a comprehensive view of tumor biology [109]. This multi-omics approach holds immense potential for improving early detection and monitoring treatment responses.

### 5.2. BEVs as Prognostic Indicators

In addition to their diagnostic utility, BEVs provide prognostic information by reflecting disease progression and therapeutic response. Specific changes in BEV composition correlate with tumor stage, metastatic potential, and treatment outcomes. In glioblastoma, longitudinal tracking of BEVs in cerebrospinal fluid has revealed dynamic changes in EGFRvIII levels, allowing clinicians to predict tumor recurrence months before imaging detects growth [86]. BEVs carrying inflammatory markers such as IL-6 and TNF-\u03b1 correlate with shorter progression-free survival, underscoring their role in glioblastoma prognosis [110]. In breast cancer, recent clinical trials have demonstrated the utility of BEV-derived miR-21 and miR-155 as stratification markers for predicting metastatic relapses in triple-negative breast cancer patients. BEVs enriched with drug resistance-associated molecules, such as P-glycoprotein, are used as prognostic indicators to guide adjuvant therapy decisions [15]. In renal cancer, circulating BEVs carrying angiogenesis-related proteins like VEGF and MMP-9 have been incorporated into risk models to predict survival outcomes in metastatic renal cell carcinoma [61]. BEV proteomic profiles are being used to identify resistance to tyrosine kinase inhibitors, helping to optimize second-line therapy choices.

Emerging technologies, such as next-generation sequencing and mass spectrometry, are being employed to profile BEVs in a high-throughput manner, facilitating their integration into clinical workflows. By tracking these biomarkers, clinicians can assess patient outcomes, monitor disease progression, and tailor therapeutic strategies accordingly [6]. Expanding our understanding of BEVs as diagnostic and prognostic tools could revolutionize cancer management by enabling personalized and precision oncology. Future studies focusing on standardizing BEV isolation and characterization methods will be crucial for their successful translation into clinical practice [22]. In addition to their diagnostic utility, BEVs provide prognostic information by reflecting disease progression and therapeutic response. Specific changes in BEV composition correlate with tumor stage, metastatic potential, and treatment outcomes. In glioblastoma, elevated levels of BEVs carrying EGFRvIII are associated with aggressive tumor behavior and resistance to standard therapies [86]. In breast cancer, BEVs containing miR-155 and miR-21 correlate with poor prognosis and increased metastatic potential [44]. In renal cancer, BEVs enriched with pro-angiogenic factors and inflammatory mediators indicate advanced disease and poor survival rates [93]. By tracking these biomarkers, clinicians can assess patient outcomes and tailor therapeutic strategies accordingly. Emerging technologies, such as next-generation sequencing and mass spectrometry, are being employed to profile BEVs in a high-throughput manner, facilitating their integration into clinical workflows.

### 5.3. BEVs as Therapeutic Agents

The therapeutic potential of BEVs lies in their ability to deliver functional molecules with high specificity and minimal toxicity [22]. Recent advancements have demonstrated their efficacy in preclinical cancer models, showcasing their versatility as delivery vehicles and immunomodulators [27].

#### 5.3.1. Drug Delivery

BEVs have been experimentally engineered to encapsulate chemotherapeutic agents, small interfering RNAs (siRNAs), and CRISPR-Cas9 components for targeted cancer therapy. In breast cancer models, BEVs loaded with doxorubicin have demonstrated targeted cytotoxicity against HER2-positive cancer cells while minimizing systemic toxicity in vitro and in vivo [111]. Similarly, siRNA-loaded BEVs targeting KRAS mutations have shown promising results in reducing tumor growth in pancreatic cancer mouse models, suggesting potential for precision medicine approaches [112]. In glioblastoma preclinical models, BEVs carrying Temozolomide improved drug penetration across the blood–brain barrier, contributing to enhanced therapeutic responses [113]. These studies underscore the promise of BEVs as novel delivery platforms, although further validation is needed for clinical translation.

#### 5.3.2. Cancer Vaccines

BEVs engineered to present tumor-associated antigens are being explored in preclinical studies as potential cancer vaccines. These vesicles have been shown to deliver antigens to antigen-presenting cells, triggering cytotoxic T-cell responses in animal models [114]. For instance, BEVs carrying tumor-specific neoantigens have been observed to activate dendritic cells and enhance anti-tumor immunity in melanoma and lung cancer mouse models [115].

#### 5.3.3. Immune Checkpoint Modulation

Recent preclinical studies have explored the potential of BEVs as delivery vehicles for immune checkpoint inhibitors within the tumor microenvironment [27]. BEVs engineered to carry anti-PD-L1 antibodies have shown the capacity to restore T-cell activity in experimental models of melanoma and non-small cell lung cancer [116]. Furthermore, combining BEV-based checkpoint delivery with traditional therapies such as radiotherapy has demonstrated synergistic effects in mouse models, including improved tumor control and extended survival [27]. However, these findings remain preliminary and require further validation before clinical translation.

#### 5.3.4. Activation of Innate and Adaptive Immunity

BEVs play a critical role in activating both innate and adaptive immune responses. They carry immunostimulatory molecules such as cytokines, lipopolysaccharides (LPS), and heat shock proteins (HSPs) [12]. BEVs carrying LPS have been shown to activate macrophages and NK cells, leading to enhanced anti-tumor activity and innate immune activation. BEVs lead to adaptive immune responses by delivering interleukins such as IL-12. BEVs have promoted T-cell activation and recruitment, improving tumor clearance in preclinical studies [117].

## 6. Challenges and Future Directions

Despite the growing body of evidence supporting the role of BEVs in cancer biology and management, several challenges must be addressed to fully realize their clinical potential. This section outlines the key limitations in BEV research and explores future directions to overcome these hurdles and translate findings into impactful applications.

### 6.1. Challenges in BEV Research

#### 6.1.1. Standardization of Isolation and Characterization Techniques

BEV isolation methods, such as ultracentrifugation and size-exclusion chromatography, often yield heterogeneous populations of vesicles, complicating downstream analyses. The lack of standardized protocols for BEV characterization hinders reproducibility and comparability across studies. Advancing methods like nanoparticle tracking analysis (NTA), flow cytometry, and single-vesicle profiling will be critical for ensuring consistency. Studies isolating BEVs using ultracentrifugation from *Escherichia coli* and *Helicobacter pylori* have reported variability in yield and purity due to differences in protocol implementation [118,119]. A lack of standardized buffer conditions resulted in contamination by non-vesicular components, complicating downstream analyses. A collaborative effort between laboratories led to the development of a consensus protocol combining ultrafiltration and density gradient ultracentrifugation, achieving reproducible results across multiple bacterial strains [16].

#### 6.1.2. Understanding BEV Heterogeneity

BEVs vary significantly in size, composition, and functional properties depending on the bacterial strain, growth conditions, and environmental factors [7]. This heterogeneity can lead to inconsistent results and reduced reproducibility across studies. Variability in BEV populations affects data interpretation, making it challenging to draw definitive conclusions about their roles in cancer progression and therapy. Studies comparing BEVs from *Escherichia coli* versus *Helicobacter pylori* demonstrate stark differences in cargo composition, influencing their interactions with host cells and tumor environments [118,119]. Inconsistent results in ovarian cancer research highlight the need for precise profiling of BEV subpopulations to distinguish between tumor-promoting and tumor-suppressing effects.

#### 6.1.3. Biases in Study Design

Over-reliance on specific bacterial strains or cancer models may skew the understanding of BEV roles in diverse clinical contexts. To mitigate biases, researchers should prioritize cross-laboratory validations using standardized protocols. Inclusion of diverse bacterial species and cancer models to better reflect the complexity of tumor–microbiome interactions. Integration of multi-omics approaches for comprehensive analyses. BEVs derived from laboratory strains of *Escherichia coli* exhibited significantly different cargo profiles compared to those isolated from clinical isolates [118]. This discrepancy skewed interpretations of their role in colorectal cancer. Cross-validation using diverse bacterial isolates and cancer patient-derived samples highlighted the need for including clinically relevant models in BEV research to ensure translational relevance.

#### 6.1.4. Clinical Translation

The scalability of BEV production and purification for therapeutic and diagnostic applications remains a major bottleneck. Regulatory hurdles related to the safety, immunogenicity, and off-target effects of BEV-based interventions need to be addressed before clinical deployment. BEVs carrying miRNAs such as miR-21 were identified as potential biomarkers for colorectal cancer [120]. However, inconsistencies in scaling isolation methods for clinical-grade BEVs delayed their validation in diagnostic trials. The adoption of microfluidic devices for large-scale BEV isolation showed promise in maintaining vesicle integrity and purity, enabling their inclusion in early-phase clinical trials for biomarker validation.

#### 6.1.5. Mechanistic Insights

While many studies have documented the involvement of BEVs in cancer progression, detailed mechanisms of their interactions with host cells and the tumor microenvironment are still poorly understood. High-resolution imaging and molecular techniques will help elucidate these interactions. BEVs from *Fusobacterium nucleatum* were shown to promote colorectal cancer metastasis through T-cell modulation [17]. However, studies lacked clarity on whether these effects were mediated by direct BEV–host cell interactions or secondary immune responses. Advanced imaging techniques, such as live-cell microscopy, demonstrated BEV internalization by T-cells, clarifying their direct role in modulating immune surveillance.

### 6.2. Future Directions

#### 6.2.1. BEVs as Precision Medicine Tools

Advances in omics technologies can enable the identification of cancer-specific BEV markers, paving the way for personalized diagnostic and therapeutic strategies. Integration of BEV-based diagnostics with liquid biopsy platforms could revolutionize early cancer detection and monitoring.

#### 6.2.2. Engineering BEVs for Therapeutic Applications

Synthetic biology approaches are enabling the precise engineering of BEVs to carry tailored cargo, such as siRNAs, CRISPR-Cas components, or immunomodulatory agents. For example, BEVs have been engineered to deliver gene-editing tools like CRISPR-Cas9 to target oncogenic mutations in vivo with high specificity [121]. Advanced hybrid systems combining BEVs with nanoparticles are being developed for enhanced drug delivery. For instance, BEV–nanoparticle hybrids have been shown to improve the stability and targeting capabilities of chemotherapeutic agents, reducing off-target toxicity while increasing therapeutic efficacy [122]. Recent breakthroughs include BEVs functionalized with peptides or aptamers for cell-specific targeting, allowing for precision delivery of therapeutics to tumor sites [123,124].

#### 6.2.3. Emerging Innovations in BEV Engineering

Programmable BEVs: DNA origami and CRISPR-Cas platforms are being used to engineer BEVs with precise cargo release mechanisms and enhanced targeting capabilities [125]. For example, BEVs programmed to release CRISPR components upon entering tumor cells have demonstrated improved specificity in preclinical models [121].

Multifunctional BEVs: Incorporating imaging agents, such as quantum dots and fluorescent dyes, into BEVs allows for simultaneous diagnostics and therapy. A recent study demonstrated BEVs carrying both doxorubicin and fluorescent markers enabled real-time tumor imaging and effective drug delivery [111].

Oncolytic BEVs: BEVs are being engineered to carry oncolytic viruses or apoptotic inducers. These vesicles selectively target tumor cells, triggering apoptosis without harming healthy tissues. For example, BEVs loaded with TRAIL (tumor necrosis factor-related apoptosis-inducing ligand) have shown promise in inducing cancer cell death in resistant tumors [126].

Hybrid Systems: Combining BEVs with synthetic nanoparticles or lipid-based carriers creates hybrid systems that enhance the bioavailability and specificity of therapeutic agents. A recent innovation integrated BEVs with gold nanoparticles for targeted photothermal therapy in melanoma models [127].

## 7. Conclusions

The field of bacterial extracellular vesicle (BEV) research is poised to make significant contributions to oncology, offering innovative solutions for cancer diagnosis, prognosis, and therapy. As versatile mediators of intercellular communication, BEVs carry a rich cargo of biomolecules that influence tumor biology through immune modulation, genetic regulation, and inflammation. Their ability to traverse biological barriers and interact with host cells underscores their translational potential as non-invasive biomarkers and targeted therapeutic agents. Despite the promising insights revealed in recent studies, challenges remain in standardizing BEV isolation and characterization techniques, scaling their clinical applications, and fully elucidating their mechanisms of action in cancer development and progression. Addressing these hurdles will require interdisciplinary collaborations and advancements in omics technologies, bioengineering, and computational tools. By integrating BEV-based diagnostics and therapeutics into the clinical landscape, we can move closer to achieving personalized and precision medicine in oncology. BEVs represent a new frontier in cancer research with the potential to revolutionize our understanding of tumor–microbe interactions and to pave the way for novel approaches to cancer management. Continued exploration of their dualistic roles in tumor promotion and suppression will not only deepen our understanding of cancer biology but also drive innovations in therapeutic strategies, ultimately improving patient outcomes. The next frontier in BEV research lies in optimizing their therapeutic application, particularly in immunotherapy and targeted drug delivery. Addressing standardization challenges and accelerating clinical validation will be pivotal for realizing their full potential in oncology.

## Figures and Tables

**Table 1:** Bacterial extracellular vesicle’s molecular cargo.

Molecular Cargo	Function in Cancer	Relevant Cancers	References
Proteins	Regulate tumor progression, immune modulation, and EMT	Breast, Lung, Ovarian, Colorectal, Gastric, and Brain	[8,12,13,14,15,16,17]
Lipids	Mediate cell signaling and influence membrane dynamics	Lung, Ovarian, Breast, and Gastric	[15,18,19,20,21]
RNA	Alter gene expression and regulate immune evasion	Ovarian, Breast, Lung, Brain, and Colorectal	[12,16,20,22,23]
DNA	Promote horizontal gene transfer and affect tumor genetics	Colorectal and Gastric	[7,14,15,24,25]
Metabolites	Modulate metabolism and influence immune suppression	Ovarian, Lung, and Gastric	[14,20,26,27,28]
Enzymes	Carries degradative enzymes such as proteases and lipases	Breast, Colorectal, Gastric, and Lung	[5,15,28,29,30]

**Table 2:** Roles of BEVs in the pathogenesis of cancers by types.

Cancer Type	Tumor Progression	Metastasis	Therapy Resistance	Key Bacterial Sources of BEVs	References
Colorectal Cancer	Promotes DNA damage and modulates immune responses	Enhances EMT and increases cell motility	BEVs transport drug efflux pumps and regulatory RNAs	*Fusobacterium nucleatum* and *Escherichia coli*	[13,56,57]
Gastric Cancer	Delivers virulence factors and induces oncogenic pathways	Remodels extracellular matrix and promotes angiogenesis	BEVs inhibit apoptosis and enhance drug efflux	*Helicobacter pylori*	[22,58]
Breast Cancer	Alters gene expression and promotes immune evasion	Facilitates EMT and tumor invasion	Modulates redox balance and activates survival pathways	*Staphylococcus aureus* and *Fusobacterium nucleatum*	[27,37,44]
Lung Cancer	Facilitates chronic inflammation and promotes oxidative stress	Forms pre-metastatic niches and increases migration	BEVs carry survival signals and downregulate immune responses	*Pseudomonas aeruginosa* and *Streptococcus pneumoniae*	[27,38,59]
Brain Cancer	Crosses the blood–brain barrier and activates oncogenic pathways	Induces angiogenesis and enhances invasiveness	Alters DNA repair mechanisms and promotes radioresistance	*Escherichia coli* and *Streptococcus pneumoniae*	[59,60]
Renal and Bladder Cancer	Triggers chronic inflammation and epithelial–mesenchymal transition (EMT)	Prepares metastatic niches and facilitates migration	Upregulates survival pathways and interferes with drug action	*Escherichia coli* (UPEC)	[22,61]
Ovarian Cancer	Induces inflammation, immune evasion, and promotes metastasis	Promotes peritoneal dissemination	Enhances chemoresistance and suppresses immune function	*Lactobacillus* spp. and *Streptococcus* spp.	[62,63,64]

## List of Abbreviations

BEVs—bacterial extracellular vesicles; EVs—extracellular vesicles; OMVs—outer membrane vesicles; TME—tumor microenvironment; miRNA—microRNA; lncRNA—long non-coding RNA; EMT—epithelial-to-mesenchymal transition; VEGF—vascular endothelial growth factor; MMPs—matrix metalloproteinases; PD-L1—programmed death-ligand 1; TLRs—Toll-like receptors; NF-κB—nuclear factor kappa B; MAPK—mitogen-activated protein kinase; siRNA—small interfering RNA; CRISPR—clustered regularly interspaced short palindromic repeats; IL-6—interleukin-6; TNF-α—tumor necrosis factor-alpha; EGFRvIII—epidermal growth factor receptor variant III; KRAS—Kirsten rat sarcoma viral oncogene; CSF—cerebrospinal fluid.
